# Parental Attitudes Toward COVID-19 Vaccination for Children: A Cross-Sectional Study Conducted in Jeddah, Saudi Arabia

**DOI:** 10.7759/cureus.88848

**Published:** 2025-07-27

**Authors:** Revan T Almalki, Lamar H Kuwaity, Shahad R Almarwan, Aasal A Alnafisi, Anfal A Felimban, Nadia El Amin, Seham Alameer

**Affiliations:** 1 College of Medicine, King Saud Bin Abdulaziz University for Health Sciences, Jeddah, SAU; 2 College of Medicine, King Abdullah International Medical Research Center, Jeddah, SAU; 3 Department of Basic Medical Science, King Saud Bin Abdulaziz University for Health Sciences College of Medicine, Jeddah, SAU; 4 Department of Basic Medical Science, King Abdullah International Medical Research Center, Jeddah, SAU; 5 College of Medicine, King Saud Bin Abdulaziz University for Health Sciences College of Medicine, Jeddah, SAU; 6 Department of Pediatrics, Ministry of National Guard Health Affairs-MNGHA), Jeddah, SAU; 7 Department of Genetic and Precision Medicine, King Abdullah Specialized Children Hospital, Jeddah, SAU

**Keywords:** children's social life, covid-19 vaccination, jeddah, parental attitude, willingness

## Abstract

Objectives: ​​​​The administration of the COVID-19 vaccine to children depends on parents’ decisions. Therefore, this study aimed to investigate parental attitudes towards COVID-19 vaccines and to assess the reasons behind their attitude and their consequences on the child's social life.

Methods: This cross-sectional study was conducted in Jeddah, Saudi Arabia, between 15^th^ April and 29^th^ August 2022, using a questionnaire distributed via social media to parents of healthy children under 18 years old. Statistical analysis was performed using JMP software (JMP Statistical Discovery LLC (part of SAS Institute), Cary, NC).

Results: A total of 562 parents participated in the survey. When asked about the likelihood of vaccinating their children, 181 (32%) answered that it was either unlikely or very unlikely. Regarding the children's social lives, 231 (88.9%) parents who had not vaccinated their children reported that their child was able to attend school regularly, socialize with friends, and travel without any concerns. Furthermore, there was a significant association between parental occupational status and children’s reception of the vaccine.

Conclusion: Although the percentage of parents who vaccinated their children was high, more than half of the parents were concerned about the vaccine’s safety. More research is needed with a larger sample and a larger geographical area to further understand the attitudes of parents towards their children’s vaccination and address their concerns. This can help governments not only in dealing with the public but also the vaccine manufacturing industry in understanding what individuals fear and are skeptical of regarding new vaccines.

## Introduction

COVID-19 is caused by SARS-CoV-2. This infectious disease originated in Wuhan, China, in 2019, while the cause of the virus is not exactly known [1]. However, it was traced back to the Huanan seafood market in Wuhan. By March 2020, the World Health Organization (WHO) declared COVID-19 a pandemic. Until November 2021, the Kingdom of Saudi Arabia had witnessed approximately 8,800 deaths due to COVID-19 [2]. Saudi Arabia was one of the first countries to implement safety measures against COVID-19 by enforcing home quarantine and travel bans both domestically and internationally [3]. The first vaccine against COVID-19 was introduced at the end of 2020, and the first FDA-approved vaccine was the Pfizer-BioNTech COVID-19 vaccine [4]. However, many individuals worldwide were skeptical about the efficacy of the vaccine. One reason for this was the spread of misinformation across social media. These included invalid data about the efficacy of the vaccine and false data about the potential side effects of the vaccine. Throughout the pandemic, conspiracy theories about the vaccine were circulating on social media worldwide. Many thought that the vaccine was created as a population control tool, especially in overpopulated or extremely poor countries. Others believed that the vaccine was fabricated for economic purposes. These conspiracy theories that had circulated online may have also caused individuals to develop mistrust and refuse the vaccine [5].

At the beginning of the pandemic, the vaccination targeted only adults and the most vulnerable groups in the community, followed by the younger generation [6]. Vaccination against COVID-19 has played a major role in controlling the pandemic and reducing the transmission [7]. However, children's tendency to receive the vaccination depended on their parents’ decisions. Hence, it was important to understand parental attitudes towards the vaccine. Therefore, this study aimed to investigate parental attitudes and perceptions towards COVID-19 vaccination and its effects on the social life of children younger than 18 years of age in Jeddah, Saudi Arabia. This research is one of the few studies to investigate this issue and provide preliminary data for policymaking and service planning.

## Materials and methods

Study design 

This was a cross-sectional research targeting parents of children aged 18 years and below living in Jeddah, Saudi Arabia.

Participants and data collection 

The participants included parents of healthy children under the age of 18 years who lived in Jeddah. The survey questionnaire was sent to the parents through diverse social media platforms (WhatsApp (Meta Platforms Inc., Menlo Park, CA), Snapchat (Snap Inc., Santa Monica, CA), Instagram (Meta Platforms Inc.), Facebook (Meta Platforms Inc.), and email) (Appendix A). A convenience sampling technique was selected to have effortless access to multiple participants, as it was possible to easily distribute it over the Internet. In addition, it was time-efficient and did not require a budget. The target sample size was 370 participants, calculated using the Raosoft online calculator (Raosoft Inc., Seattle, WA) with an estimated population of 100,000. While calculating the sample size, a margin of error of 5%, a confidence level of 95%, and an expected prevalence of 50% were used. Data were collected between April 15 and August 29, 2022, resulting in a total of 1,021 responses, 562 of which were from individuals living in Jeddah and were included in the final research.

To ensure confidentiality, a consent form was included at the beginning of the questionnaire, and no personal information was collected to protect participants’ privacy. Moreover, all participants were provided with the option of withdrawing their participation. The questionnaire was carefully designed to avoid suggestive or biased wording in order to minimize response bias.

Development of the questionnaire

The study utilized a questionnaire that had been previously developed, tested, and validated in both Arabic and English by Altuhaifi et al. (2021), ensuring the reliability of the data collection tool [5]. Extra questions were added to the questionnaire by our research group (one pediatric consultant, one assistant professor, and six medical students) due to the addition of a secondary objective. This secondary objective aimed to evaluate how parental attitudes toward the COVID-19 vaccine might influence their decision to vaccinate their children and how such vaccination status could, in turn, affect the children’s social interactions. The survey was checked for face validity (it is a subjective judgment of a test to check if it can measure the intended variables) by two experts from our research unit and sent to friends and family members. Additional questions were also reviewed by two professors for face validity and relevance. The questionnaire was modified according to the reviewers’ comments. Furthermore, the responses from the four participants were not included in the published survey and were excluded after the questionnaire was modified. To conclude, the next sections explain more about the items analyzed in each part of the questionnaire. 

Background characteristics

Demographic variables such as gender, age, relationship status (e.g., married, divorced, widowed), occupational status, and educational level were collected. Also, information about the age of the children was collected, as it could influence parental decisions regarding vaccination. Then, more questions were intended to examine the reasons behind the parents’ willingness to vaccinate their children, their opinions about statements related to the COVID-19 vaccine, frequency of exposure to information related to COVID-19 vaccination on social media, and frequency of following safety precautions. Finally, questions were added to assess the effect of parental attitudes towards the COVID-19 vaccine on children’s social lives [8].

Parental acceptability of free COVID-19 vaccination 

The participants were asked a question: “COVID-19 vaccines are now available for children. What is your likelihood of having your child under the age of 18 years take up the free COVID-19 vaccination provided by the government?” Responses were subdivided into five categories, namely, "Very unlikely", "Unlikely", "Neutral", "Likely", and "Very likely".

Assessment of parental perceptions regarding COVID-19 vaccination based on the Theory of Planned Behavior (TPB)

Constructed statements were given to the participants to understand their perceptions. These statements were constructed based on the TPB [8]. An example of the statement is “A COVID-19 vaccination is highly effective in protecting your child from COVID-19,” and the response options were (disagree, neutral, agree). In another section, the participants were presented in two tables: the first table explored the reasons behind parents’ acceptance of the vaccine, which included protection of vaccinated children, protection of other unvaccinated people, governmental recommendations, vaccine and COVID-19 infection risk, parents’ knowledge about COVID-19 vaccine effectiveness, vaccine’s risks and benefits, and role of vaccine in return to normal life. The second table was intended to examine the reasons behind their disapproval of the vaccine; the section explored the availability of adequate trials and evidence of the vaccine’s safety, its effects on children, children’s risk of contracting COVID-19, vaccine efficacy, children’s history of COVID-19 infection, and parents’ lack of knowledge about the vaccine. Both tables had the response categories of ("Yes" or "No").

Influence of social media

This section of the questionnaire analyzed the frequency of exposure to COVID-19-related information on different social media platforms (WhatsApp, Twitter, Snapchat, TikTok, etc.). This section contained four questions; each question consisted of four multiple-choice options (“Almost never,” “Seldom,” “Sometimes,” and “Always”). The parents were asked to report the extent of exposure to positive information related to COVID-19 vaccination (for example, “New vaccines entering clinical trials, promising efficacy of the vaccines, and vaccines that will enter the market soon”), negative information related to COVID-19 vaccination (for example, concerns about efficacies and supplies, side-effects of the vaccines, and that receiving the vaccines will cause COVID-19), testimonials given by participants of the COVID-19 vaccine clinical trials, and negative information about some vaccines in Saudi Arabia (for example, giving problematic vaccines and severe side effects) [8].

Assessing the effect of parental attitude on children’s social life

The participants were asked seven questions to assess the effect of receiving or not receiving the COVID-19 vaccination on their children’s social lives. First, parents were asked if their children had been vaccinated or not with a “Yes” or “No” type of question. 

Depending on the parents’ answers, they were transferred to a specific section of the survey to study the effects of their decisions on their children’s social lives. The statements included in both tables aimed to evaluate the children’s ability to attend school regularly, their absence due to having or not having the vaccine, their ability to hang out with friends normally as often as they used to before the vaccine was imposed, and their ability to have good social relationships. In addition, they were asked about any improvements in their children’s social lives after receiving or not receiving the COVID-19 vaccine, their ability to travel without any concerns, and whether the children were able to spend time out as usual.

Data analysis

Microsoft Excel (Microsoft Corp., Redmond, WA) was used to organize the data, and JMP Pro 15 software (JMP Statistical Discovery LLC (a subsidiary of SAS Institute), Cary, NC) was used to analyze data and identify associations. The chi-square test was used to determine the associations between the variables, and a p-value of less than or equal to 0.05 was used to determine significance (P-values ≤ 0.05).

Ethical consent

This study was approved by the Institutional Review Board (IRB) of King Abdullah International Medical Research Center (KAIMRC) on 9^th^ March 2022 (approval number: IRB/0476/22).

The authors obtained consent from all the participants to report their clinical information to the journal. All participants understood that their anonymity would be maintained to ensure data confidentiality and privacy.

## Results

Table 1 describes the parents’ demographics. Many parents (326, 58%) were between 36 and 50 years old. The sample included 277 females (49.3%) and 285 males (50.7%). The number of married participants was significantly high: 528 (94%). In addition, most parents who completed the survey were college undergraduates (372, 66.2%), and many were employed (341, 60.7%).

**Table 1:** Demographics of the parents in the study group Descriptive data are represented as N (%).

Age (years)	N (%)
36-50	326 (58)
Older than 50	138 (24.6)
26-35	83 (14.8)
18-25	15 (2.7)
Gender	N (%)
Male	285 (50.7)
Female	277 (49.3)
Relationship status	N (%)
Married	528 (94)
Divorced	19 (3.4)
Single	10 (1.8)
Widowed	5 (0.9)
Educational level	N (%)
University undergraduate	372 (66.2)
High school or less	104 (18.5)
Postgraduate	86 (15.3)
Occupational status	N (%)
Currently working	341 (60.7)
Retired/unemployed	221 (39.3)

There was a question to assess parents’ acceptance of vaccination for their children who were still under the age of 18 years. Approximately one-third of the participants answered either “Unlikely” or “Very unlikely,” 181 (32%). However, the majority (291, 52%) were either “Likely” or “Very likely” to vaccinate their children.

When participants were asked to describe their adherence to different COVID-19 precautions and preventive measures in the last month, more than half of the parents (296, 52.7%) wore masks in public places and transport, and 247 (43.95%) of participants wore masks whenever they were in close contact with other people at their workplace. In contrast, fewer parents (24, 4.3%) never wore masks in public places and transportation. A small percentage (41, 7.3%) never wore masks when they were in close contact with other people in their workplace. In addition, 272 (48.4%) of the parents sanitized their hands every time after returning from public places or touching public installations. Many participants (297, 53%) did not avoid social gatherings in the past month.

Table 2 describes the reasons for parents to vaccinate their children against COVID-19. Most participants (414, 73.7%) chose to vaccinate their children because it was recommended by the government. Moreover, more than 50% of the parents agreed with the different statements mentioned in Table 2. 

**Table 2:** Reasons for parents to vaccinate their children against COVID-19 Data are represented as N (%). Statistical comparisons were performed using the chi-square test. P-values <0.0001 were considered statistically significant.

I will vaccinate my children with the COVID-19 vaccine because...	N (%)		χ²-value	P-value
	Yes	No		
The government recommends it.	414 (73.7)	148 (26.3)	562	<0.0001
I want to return to normal life.	373 (66.4)	189 (33.6)	226.4	<0.0001
It protects my children.	352 (62.6)	210 (37.4)	224.5	<0.0001
My children are at risk of being infected with COVID-19.	347 (61.7)	215 (38.3)	203.4	<0.0001
The benefits outweigh the risks.	340 (60.5)	222 (39.5)	180.2	<0.0001
I believe in the effects of vaccination.	338 (60.1)	224 (39.9)	203.9	<0.0001
It protects others.	322 (57.3)	240 (42.7)	193.2	<0.0001

Table 3 describes the reasons why parents did not vaccinate their children against COVID-19. Many of the parents (304, 54.1%) were concerned about the vaccine’s safety. However, most parents had opposing responses for the following factors: lack of evidence, minimal viral effects on children, absence of risk of infection, lack of effectiveness, previous COVID-19 infection, and lack of information about the vaccine.

**Table 3:** Reasons why parents did not vaccinate their children against COVID-19 Data are represented as N (%). Statistical comparisons were performed using the chi-square test. P-values <0.0001 were considered statistically significant.

I will NOT vaccinate my children with the COVID-19 vaccine because...	N (%)		χ²-value	P-value
	Yes	No		
I am concerned about the vaccine's safety.	304 (54.1)	258 (45.9)	562	<0.0001
There is not enough evidence.	277 (49.3)	285 (50.7)	259.6	<0.0001
I have no information about the vaccine.	244 (43.4)	318 (56.6)	220.8	<0.0001
My children are not at risk.	213 (37.9)	349 (62.1)	174.8	<0.0001
The virus hardly affects children.	205 (36.5)	357 (63.5)	174.4	<0.0001
My children were already infected with the COVID-19 virus.	201 (35.8)	361 (64.2)	113.3	<0.0001
I think it is not effective.	192 (34.2)	370 (65.8)	213.6	<0.0001

Regarding parents’ agreement with statements related to COVID-19 vaccination. The ability of the country to provide an adequate vaccine supply received the highest agreement among participants (415, 74%). Only 26 (4.8%) of participants disagreed with this statement. The least agreement (38, 6.9%) was associated with the parents’ inability to vaccinate their children due to a lack of time, making this statement the most disagreed upon by 423 (75.3%) of participants. Approximately 256 (45.7%) of participants were either neutral or uncertain regarding the protection duration of the vaccine.

Several rumors about the vaccine have been published on social media platforms and spread among citizens. Despite this, most parents answered “sometimes” when asked about their exposure to any vaccine-related information on social media. Our findings were not consistent with Viswanath et al.’s results (2021) [9]. Although they reported that the participants chose social media as the main source of health-related information or anti-vaccine news, our research showed no significant effect of social media usage on the likelihood of getting vaccinated.

Table 4 shows the frequency of parental exposure to information related to the COVID-19 vaccine on social media. Most parents (260, 46%) answered “Sometimes” to being exposed to positive information, and 246 (43.7%) answered “Sometimes” to being exposed to negative information related to COVID-19 vaccination. Moreover, a substantial number of parents (231, 41.1%) answered “Sometimes” about being exposed to testimonies given by participants of the COVID-19 vaccine trial. In addition, many parents answered “Sometimes” on being exposed to negative information about some vaccines in Saudi Arabia; on the contrary, few answered “always” (47, 8.4%).

**Table 4:** Frequency of parental exposure to information related to the COVID-19 vaccine on social media Descriptive data are represented as N (%).

Frequency of parental exposure to the following statements	N (%)				
	Always	Sometimes	Seldom		Almost never
Positive information related to COVID-19 vaccination.	80 (14.2)	260 (46.3)	112 (19.9)		110 ( 19.6)
Negative information related to COVID-19 vaccination.	95 (16.9)	246 (43.8)	138 (24.6)		83 (14.8)
Testimonials given by participants of the COVID-19 vaccine trial.	68 (12.1)	231 (41.1)	166 (29.5)		97 (17.3)
Negative information about some vaccines in Saudi Arabia.	47 (8.4)	197 (35.05)	170 (30.25)		148 (26.3)

More than half of the parents answered “Yes” to getting their child vaccinated. Table 5 illustrates the effects on the child's social life when the parents decided to vaccinate them. A total of 290 (96%) of parents who vaccinated their child agreed that their child was able to attend school regularly. Moreover, 282 (93.4%) of parents acknowledged that their children had good social relationships. The majority (243, 80.5%) answered "Yes" to their child being able to travel without any concerns. Only the children of 101 (33.4%) parents were not able to socialize with friends normally and as often as they used to before the vaccine was imposed, unlike the remaining 201 (66.6%).

**Table 5:** The effects of receiving the COVID-19 vaccine on the child's social life Descriptive data are represented as N (%).

Statements about receiving the COVID-19 vaccine on the child's social life	N (%)		
	Yes	No	
My child is able to attend school regularly.	290 (96)	12 (4)	
My child has good relationships.	282 (93.4)	20 (6.6)	
My child is able to spend time out as usual.	254 (84.1)	48 (15.9)	
My child is able to travel without any concerns.	243 (80.5)	59 (19.5)	
My child did not have any absences due to receiving the COVID-19 vaccine.	215 (71.2)	87 (28.8)	
My child is able to hang out with friends normally and as often as they used to see them before the vaccine was imposed.	201 (66.6)	101 (33.4)	
I did not notice any improvement in my child's social relationships, even though he took the COVID-19 vaccine.	121 (40.1)	181 (59.9)	

Table 6 presents the effects of not receiving the COVID-19 vaccine on the child's social life, according to parents. Only 36 (13.85%) of parents agreed that their children were unable to attend school regularly, while 92 (35.4%) of parents stated that their children were not able to socialize with friends normally as often as they used to before the vaccine. However, 231 (88.85%) of parents admitted that their children still had good social relationships.

**Table 6:** The effects of not receiving the COVID-19 vaccine on the child's social life Descriptive data are represented as N (%).

Statements about NOT receiving the COVID-19 vaccine on the child's social life	N (%)		
	Yes	No	
My child has good relationships.	231 (88.85)	29 (11.15)	
My child is able to attend school regularly.	224 (86.15)	36 (13.85)	
My child is able to spend time out as usual.	208 (80)	52 (20)	
My child is able to travel without any concerns.	202 (77.7)	58 (22.3)	
My child did not have any absences due to not receiving the COVID-19 vaccine.	172 (66.15)	88 (33.85)	
My child is able to hang out with friends normally and as often as they used to see them before the vaccine was imposed.	168 (64.6)	92 (35.4)	
I did not notice any improvement in my child's social relationships, even though he didn’t take the COVID-19 vaccine.	107 (41.15)	153 (58.85)	

Factors associated with parental acceptability of COVID-19 vaccination

Parents’ Occupational Status 

There was a significant association between the occupation of parents and the vaccination of their children (p<0.0001). Many parents (180, 53%) who were currently working reported that their child did not receive the vaccine. However, most unemployed parents (141, 64%) agreed to get their children vaccinated. 

Age of Parents

The older the parents, the more likely they were to vaccinate their children (p-value < 0.0001). Most parents between 18 and 35 years refused to get their child vaccinated, unlike those who were older than 35 years.

Likelihood 

As expected, the administration of the vaccine to children was influenced by parental tendencies (p<0.0001). Most of the parents who answered that they were “Likely” to vaccinate vaccinated their children.

Occupational Status and Likelihood 

Unemployed parents were more likely to vaccinate their children than those who were currently working (p=0.007). The proportion of parents who answered “Unlikely” or “Neutral” was higher among working parents.

## Discussion

Literature review

Multiple studies related to factors that affect parents’ attitudes toward COVID-19 and other vaccines were analyzed and summarized as follows.

Factors Affecting Acceptability 

A national study conducted by Szilagyi et al. (2021) demonstrated that the likelihood of children getting vaccinated was greater among parents of older children as well as among parents with a bachelor’s degree or higher education [10].

Factors Affecting Hesitancy 

Numerous factors can affect parental hesitancy. According to Olusanya et al. (2021), these factors included concerns about vaccine costs, religious beliefs, urban and rural residencies, and other social determinants of health (SDoH) [11]. In 2022, a cross-sectional study conducted in Jordan emphasized that more than half of the parents were unwilling to get their children vaccinated [12]. Their decision was influenced by many factors, similar to the findings of Olusanya et al. [11, 12].

In 2022, Kaidi et al. described the perception and hesitancy of parents towards having their children vaccinated against COVID-19 [13]. Parents of children who are 18 years old and younger were recruited by the University of Southern California (USC) to assess their perception using a questionnaire based on a modified Vaccine Hesitancy Scale developed by the WHO’s Strategic Advisory Group of Experts (SAGE). Overall, vaccine hesitancy and risk perception increased during the pandemic. 

Side Effects of Vaccines

The side effects and safety concerns of COVID-19 vaccines are among the most common causes of hesitation. A US study found that most uncertainty about the vaccine originated from doubts regarding its safety [14]. The parents who were confident in terms of vaccine security and lack of side effects were 17% more likely to administer it to their children.

A study conducted in Saudi Arabia found that a total of 74% of the participants were hesitant to get their children vaccinated against COVID-19, and 80% of the children did not receive the vaccination. More than half of the patients who did not receive the vaccine reported side effects as their main concern [15].

A similar survey evaluated the willingness of parental COVID-19 vaccination for children aged 5-11 years with chronic conditions in Italy. Since this research was conducted in the context of a large pandemic, parents were unwilling to provide the vaccine to their children owing to either a lack of knowledge or fear of adverse effects. Research has suggested that parental education can improve acceptance of vaccination and reduce the spread of this infectious disease [16].

General Knowledge about Vaccines

To assess vaccination hesitancy, a study was conducted in a Colorado healthcare organization to test whether adequate understanding and knowledge about a vaccine would affect parental attitudes towards it. Results revealed that parents with sufficient information regarding the vaccine showed a more positive approach [17]. Another study reported similar results regarding parental knowledge. Bauer et al. (2021) in Pöggstall, Austria, assessed the attitudes of adults (n=295) and their children (n=316) towards general vaccinations as well as mandatory vaccinations [17]. Regarding nationally recommended vaccinations, 56.6% of the adults had a positive attitude, 67% of whom were 60 years old or older. Most of the adults (73.6%) who had a positive attitude towards vaccination had tertiary education (the education one receives after high school) [18]. Participants with skeptical or negative attitudes were less likely to obtain high knowledge scores [18]. Another interesting finding in this study was that younger participants had a more negative attitude, and more than half of the participants aged 60 years and above had a positive attitude towards vaccination [18].

In a similar study conducted in Malaysia, researchers focused on the role of religious beliefs and fear of illness in vaccine skepticism [19]. They assessed the knowledge and attitudes of 200 postnatal mothers towards vaccination, and the majority (73.5%) had excellent knowledge and, hence, a brighter outlook towards it. However, in this study, 23% were hesitant because of misconceptions about the association between vaccination and autism. Also, out of the mothers, 4% still had religious misconceptions.

In Riyadh, Saudi Arabia, a survey of 325 participants evaluated the effects of social media on parental attitudes towards vaccinations; 89% of the parents were not hesitant about vaccinating their children, while 11% were hesitant, but the reason was not associated with their level of education or social media use [20].

Studies that have assessed parental attitudes towards influenza vaccines show that parents’ attitudes towards vaccination ranged from hesitancy to confidence. Hamadah et al. studied parental attitudes and beliefs towards seasonal influenza vaccination in Riyadh, Saudi Arabia [21]. Out of 388 parents, 76.8% were not hesitant in getting their children vaccinated. However, 61.86% did not get their children vaccinated. The most common reason behind the parents’ decisions was their belief that the vaccine was unnecessary (36.2%) [21]. Similarly, a study conducted in Turkey evaluated the attitudes of parents of high socioeconomic status toward influenza vaccination [22]. Out of 285 participants, only 8.8% of parents had their children vaccinated, leaving 260 children unvaccinated; the reason reported by 64 participants (24.9%) was that the vaccination was unnecessary, a conclusion similar to that of Hamadah et al. [21,22].

In addition, two other studies on influenza and hepatitis B vaccines revealed an association between higher education and knowledge of the disease, with a positive attitude towards vaccinations [23]. Another study with the same design was conducted in 2005 by Dannetun et al. to illustrate parents' attitudes towards hepatitis B vaccination in children [24].

Since the start of the pandemic, many companies have started manufacturing vaccines for testing. When the first COVID-19 vaccine was administered, most citizens were skeptical because of what they claimed was the rapid process of analysis and administration. Adult immunization was the main purpose of vaccination; however, research is progressing, and technology is constantly evolving. As a result, current vaccinations cover younger age groups too. Because parents were doubtful about the COVID-19 vaccine, rumors and questions began to arise on social media platforms. Consequently, this study attempted to understand and recognize the factors that affected the parents' decisions and led them to either approve or disapprove of the vaccine.

Understanding parental attitudes towards vaccines is crucial for assessing the factors that influence their decisions and addressing them. Although 271 (48%) of participants answered either “Unlikely,” “Very unlikely,” or “Neutral” to their likelihood of vaccinating their children, 302 (54%) of the participants administered the vaccine. A possible reason for these findings is the Ministry of Health’s declaration regarding the vaccine as being mandatory for all citizens. This might suggest a powerful impact of governmental decisions on parents’ acceptance of the vaccine, making it a convenient method for advising the population about new remedies or preventions, if applicable. 

Most participants in our study chose concerns over the vaccine’s safety as the main reason for refusing it. Similarly, Omer et al. (2009) identified vaccine safety as a major cause of vaccine rejection [25]. The rapid manufacturing and approval of the COVID-19 vaccine might be the reason for parents’ concerns regarding its safety. Educating the community and providing reliable health-related information could increase assurance and minimize doubts. 

Some other intriguing and unexpected findings in this study need to be explained. Surprisingly, parents’ choice to vaccinate their children did not have any significant effect on the children’s social lives. This raises the question of whether COVID-19-related regulations were strict enough not to see the anticipated results regarding quality of life.

Recommendations and Limitations 

Although the study’s findings were significant, some key limitations must be highlighted. First, conducting the study in a specific city, Jeddah, may limit the generalizability of the findings. Moreover, a sample size of 562 participants was sufficient for the assumption, but not to base a strong generalization. Furthermore, convenience sampling was used to collect the data, which increased the chance of biased results, mainly selection bias. An internet-based survey was distributed among parents using social media because of a lack of time and budget. However, some parents had no access to social media platforms, which might contribute to the inability of this sample to represent the entire population of parents living in Jeddah. Some questions in the survey are not very clear to the average population (parents), and only educated/healthcare professionals can answer them, which may influence the responses to the questions. Another limitation is that the participants were able to complete the survey more than once. Since parental decisions regarding the COVID-19 vaccine can play a great role in improving vaccination rates among children, there is a critical need to plan future campaigns to increase awareness, enhance positive attitudes, and improve behavioral control. Additionally, more studies should assess and analyze this relationship and investigate the future effects of parents’ attitudes on children’s vaccination status.

## Conclusions

While assessing parents’ attitudes towards administering the COVID-19 vaccine to their children, the side effects and safety of the vaccine were among the most common factors for hesitancy. This outcome is consistent with previous studies. A clear analysis of the factors influencing parents’ attitudes can cause a huge shift not only in how governments deal with the public but also in the vaccine manufacturing industry. If manufacturing companies can understand what individuals fear and are skeptical of when it comes to new vaccines, they can address those concerns in commercial campaigns and attain an advantage when competing in the market.

## Appendices

Appendix A 

Questionnaires Sent to the Participants

**Table 7:** Parental Demographics and Attitudes Toward COVID-19 Vaccination for Children Under 18 Years

1. Do you have any child under the age of 18 years old?							
No				Yes			
If Yes, Please answer the following questions: 2.How many of your children are under the age of 18							
1		2		3		4 or more	
3. How old is your child who is under the age of 18 years ( you may select more than one answer ) ?							
(0-4)		(5-9)		(10-13)		(14-17)	
4. COVID-19 vaccines are likely to become available for children. What is your likelihood of having your child under the age of 18 years take up free COVID-19 vaccination provided by the government?							
Very unlikely	Unlikely		Neutral		Likely		Very likely

**Table 8:** Knowledge about COVID-19 vaccine assessment (True vs False)

1. Do you agree with the following statements related to COVID-19 vaccination?	Disagree	Neutral	Agree
A. COVID-19 vaccination is highly effective in protecting your child from COVID-19			
B. Taking up COVID-19 vaccination can contribute to the control of COVID-19 in Saudi Arabia			
C. Saudi Arabia will have adequate supply of COVID-19 vaccination			
D. Your child will have severe side effects after receiving COVID- 19 vaccination			
E. The protection of COVID-19 vaccines will only last for a short time			
F. Your child is afraid of vaccination			
G. You do not have time to take your child for COVID-19 vaccination			
H. Your family member would support you in having your child take up COVID-19 vaccination			
I. Having the child receive COVID-19 vaccination is easy for you if you want them to			

**Table 9:** Reasons for taking COVID-19 vaccination

2. What is the reason that you will vaccinate your child ?	YES	NO
I will vaccinate my children with COVID-19 vaccine because it protects them.		
I will vaccinate my children with COVID-19 vaccine because it protects the others.		
I will vaccinate my children with COVID-19 Vaccine because the government recommends it.		
I will vaccinate my children with COVID-19 vaccine because they are in risk of being infected with COVID-19.		
I will vaccinate my children with COVID-19 vaccine because they are in risk of being infected with COVID-19.		
I will vaccinate my children with COVID-19 vaccine because I believe in the effect vaccinations.		
I will vaccinate my children with COVID-19 vaccine because the benefits outweigh the risks.		
I will vaccinate my children with COVID-19 vaccine to return to normal life.		

**Table 10:** Reasons for not taking COVID-19 vaccination

3. What is the reason that you will not vaccinate your child ?	YES	NO
I will NOT vaccinate my children with COVID-19 vaccine because there is no enough evidence.		
I will NOT vaccinate my children with COVID-19 vaccine because I am concern about its safety.		
I will NOT vaccinate my children with COVID-19 vaccine because it is hardly affect children.		
I will NOT vaccinate my children with COVID-19 vaccine because they are not at risk.		
I will NOT vaccinate my children with COVID-19 vaccine because I think it is not effective.		
I will NOT vaccinate my children with COVID-19 vaccine they already had COVID-19.		
I will NOT vaccinate my children with COVID-19 vaccine because I have no information about the vaccine.		

**Table 11:** Frequency of exposing to information related to COVID-19 vaccination on social media in the past month

3. How frequent you were exposed to the following information related to COVID-19 vaccination on (WhatsApp, Twitter, Snapchat, Tiktok, etc.) in the last month ?	Almost never	Seldom	Sometimes	Always
A. Positive information related to COVID-19 vaccination (e.g., new vaccines entering clinical trials, promising efficacy of the vaccines, and vaccines, and vaccines will enter the market soon)	0	1	2	3
B. Negative information related to COVID-19 vaccination (e.g., concerns about efficacies and supplies, side-effects of the vaccines, and receiving vaccines will cause COVID-19)	0	1	2	3
C. Testimonials given by participants of the COVID- 19 vaccine clinical trials	0	1	2	3
D. Negative information about some vaccines in Saudi Arabia (e.g., giving problematic vaccines and severe side effects)	0	1	2	3

**Table 12:** Frequency of following safety measures in the past month

3. How frequent you performed the following safety measures in the last month ?	Every time	Often	Sometimes	Never
A. Facemask wearing in public places/transportations other than workplaces in the past month				
B. Facemask wearing when you have close contact with other people in workplace in the past month				
C. Sanitizing hands (using soaps, liquid soaps or alcohol-based sanitizer) after returning from public spaces or touching public installation				

**Table 13:** Social distancing assessment

7. In the past month did you…	Yes	No
A. Avoid social/meal gathering with other people who do not live together		
B. Avoid crowed places		

**Table 14:** Sociodemographic Characteristics and Health-Related Practices of Participants

1. How old are you?														
18-25 years			26-35 years					36-50 years				Older than 50years		
2. What is your gender?														
Male								Female						
3. What is your relationship status?														
Single			Married					Divorced				Widowed		
4. What is your education level?														
rimary school or below	middle/intermediate school				secondary/ high school			College			University			Postgraduate
5. What is your occupational status?														
Employee of the Healthcare sector		Employee in other sectors				Freelancer			Unemployed				Retired	
6. Have you ever-received seasonal influenza vaccination?														
No				Yes, within one year						Yes, beyond one year ago				
7. Do you have a family member with history of COVID-19?														
Yes							No							

## References

[1] Koopmans M, Daszak P, Dedkov VG (2021). Origins of SARS-CoV-2: window is closing for key scientific studies. Nature.

[2] Saudi Arabia: WHO; 2021 (2021). COVID-19 Cases, World. https://data.who.int/dashboards/covid19/cases?n=c.

[3] Algaissi AA, Alharbi NK, Hassanain M, Hashem AM (2020). Preparedness and response to COVID-19 in Saudi Arabia: building on MERS experience. J Infect Public Health.

[4] (2022). FDA approves first COVID-19 vaccine. https://www.fda.gov/news-events/press-announcements/fda-approves-first-covid-19-vaccine.

[5] Altulaihi BA, Alaboodi T, Alharbi KG, Alajmi MS, Alkanhal H, Alshehri A (2021). Perception of parents towards Covid-19 vaccine for children in Saudi population. Cureus.

[6] Ritchie H, Mathieu E, Rodés-Guirao L (2021). Saudi Arabia: Coronavirus pandemic country profile. https://ourworldindata.org/coronavirus/country/saudi-arabia.

[7] Oordt-Speets A, Spinardi J, Mendoza C, Yang J, Morales G, McLaughlin JM, Kyaw MH (2023). Effectiveness of COVID-19 vaccination on transmission: a systematic review. COVID.

[8] Zhang KC, Fang Y, Cao H (2020). Parental acceptability of COVID-19 vaccination for children under the age of 18 years: cross-sectional online survey. JMIR Pediatr Parent.

[9] Viswanath K, Bekalu M, Dhawan D, Pinnamaneni R, Lang J, McLoud R (2021). Individual and social determinants of COVID-19 vaccine uptake. BMC Public Health.

[10] Szilagyi PG, Shah MD, Delgado JR (2021). Parents’ intentions and perceptions about COVID-19 vaccination for their children: results from a national survey. Pediatrics.

[11] Olusanya OA, Bednarczyk RA, Davis RL, Shaban-Nejad A (2021). Addressing parental vaccine hesitancy and other barriers to childhood/adolescent vaccination uptake during the coronavirus (COVID-19) pandemic. Front Immunol.

[12] Abuhammad S, Khader Y, Hamaideh S (2022). Attitude of parents toward vaccination against COVID-19 for own children in Jordan: a cross-sectional study. Inform Med Unlocked.

[13] He K, Mack WJ, Neely M, Lewis L, Anand V (2022). Parental perspectives on immunizations: impact of the COVID-19 pandemic on childhood vaccine hesitancy. J Community Health.

[14] Thunström L, Ashworth M, Finnoff D, Newbold SC (2021). Hesitancy Toward a COVID-19 Vaccine. Ecohealth.

[15] Alghamdi S (2022). The attitude of parents toward their children receiving the COVID-19 vaccine. Children (Basel).

[16] Bianco A, Della Polla G, Angelillo S, Pelullo CP, Licata F, Angelillo IF (2022). Parental COVID-19 vaccine hesitancy: a cross-sectional survey in Italy. Expert Rev Vaccines.

[17] Ruiz JB, Bell RA (2022). Parental COVID-19 vaccine hesitancy in the United States. Public Health Rep.

[18] Bauer A, Tiefengraber D, Wiedermann U (2021). Towards understanding vaccine hesitancy and vaccination refusal in Austria. Wien Klin Wochenschr.

[19] Balbir Singh HK, Badgujar VB, Yahaya RS (2019). Assessment of knowledge and attitude among postnatal mothers towards childhood vaccination in Malaysia. Hum Vaccin Immunother.

[20] Al-Regaiey KA, Alshamry WS, Alqarni RA (2022). Influence of social media on parents' attitudes towards vaccine administration. Hum Vaccin Immunother.

[21] Hamadah RE, Hussain AN, Alsoghayer NA, Alkhenizan ZA, Alajlan HA, Alkhenizan AH (2021). Attitude of parents towards seasonal influenza vaccination for children in Saudi Arabia. J Family Med Prim Care.

[22] Gündüz S, Yüksel NC, Aktoprak HB, Canbal M, Kaya M (2014). Attitudes towards influenza vaccination in high socioeconomic status Turkish parents. Turk J Med Sci.

[23] Thanee C, Kittikraisak W, Sinthuwattanawibool C (2021). Knowledge, attitude/perception, and practice related to seasonal influenza vaccination among caregivers of young Thai children: a cross-sectional study. PLoS One.

[24] Dannetun E, Tegnell A, Giesecke J (2007). Parents' attitudes towards hepatitis B vaccination for their children. A survey comparing paper and web questionnaires, Sweden 2005. BMC Public Health.

[25] Omer SB, Salmon DA, Orenstein WA, deHart MP, Halsey N (2009). Vaccine refusal, mandatory immunization, and the risks of vaccine-preventable diseases. N Engl J Med.

